# Short-term effectiveness of precise safety decompression via double percutaneous lumbar foraminoplasty and percutaneous endoscopic lumbar decompression for lateral lumbar spinal canal stenosis: a prospective cohort study

**DOI:** 10.1186/s12891-021-03956-9

**Published:** 2021-01-14

**Authors:** Yu Wang, Mingyan Deng, Hao Wu, Ye Wu, Chuan Guo, Dongfeng Zhang, Qingquan Kong

**Affiliations:** 1 Department of Orthopedic Surgery, National Clinical Research Center for Geriatrics, West China Hospital, Sichuan University, Chengdu, 610041 Sichuan Province China; 2 WestChina-California Research Center for Predictive Intervention Medicine, West China Hospital, Sichuan University, Chengdu, China

**Keywords:** Lumbar lateral spinal canal, Double percutaneous lumbar foraminoplasty, Percutaneous endoscopic lumbar decompression

## Abstract

**Purpose:**

This prospective cohort study reports on a modified technique, namely precise safety decompression via double percutaneous lumbar foraminoplasty (DPLF) and percutaneous endoscopic lumbar decompression (PELD) for lateral lumbar spinal canal (LLSC) stenosis, and its short-term clinical outcomes.

**Methods:**

The study analyzed 69 patients with single-level LLSC stenosis simultaneously occurring in both zones 1 and 2 (defined as retrodiscal space and upper bony lateral recess respectively by new LLSC classification) who underwent DPLF–PELD from November 2018 to April 2019. Clinical outcomes were evaluated according to preoperative, 3 months postoperatively, and last follow-up, via leg pain/low back pain (LBP) visual analog scale (VAS) scores, Oswestry disability index (ODI) scores, and the Macnab criteria. The postoperative MRI and CT were used to confirm the complete decompression, and flexion-extension x-rays at the last follow-up were used to observe lumbar stability.

**Results:**

All patients successfully underwent DPLF–PELD, and the stenosis was completely decompressed, confirmed by postoperative MRI and CT. The mean follow-up duration was 13 months (range: 8–17 months). The mean preoperative leg pain VAS score is 7.05 ± 1.04 (range 5–9), which decreased to 1.03 ± 0.79(range: 0–3) at 3 months postoperatively and to 0.75 ± 0.63 (range: 0–2) by the last follow-up visit (*p* < 0.05). The mean preoperative ODI was 69.8 ± 9.05 (range: 52–85), which decreased to 20.3 ± 5.52 (range: 10–35) at the third month postoperatively and to 19.6 ± 5.21 (range: 10–34) by the final follow-up visit (*p* < 0.05). The satisfactory (excellent or good) results were 94.2%. There was one patient with aggravated symptoms, which were relieved after an open surgery. Two patients had a dural tear, and two patients suffered postoperative LBP. No recurrence or segmental instability was observed at the final follow-up.

**Conclusion:**

DPLF–PELD could be a good alternative for the treatment of LLSC stenosis patients whose stenosis occurred in both zones 1 and 2.

**Trial registration:**

Chinese Clinical Trial Registry (ChiCTR1800019551). Registered 18 November 2018.

**Supplementary Information:**

The online version contains supplementary material available at 10.1186/s12891-021-03956-9.

## Introduction

Along with the aging of society, the incidence of degenerative lumbar disorders has increased, becoming one of the main reasons for lumbar surgery in elderly patients [1, 2]. Owing to the remarkable evolution of percutaneous endoscopic lumbar decompression (PELD), the application of spinal endoscopy is shifting from the treatment of soft disk herniation to complex lumbar spinal stenosis. Satisfactory results of PELD in treating lumbar spinal stenosis (LSS) are reported at 82–92% [3].

It has been widely accepted that LSS anatomically involved the central canal, lateral recess, foramen, or any combination of these locations [4, 5]. However, the concept of a lateral recess still has no universal definition, and was frequently represented by other ambiguous terms, such as radicular canal, lateral recess zone or nerve root canal [5–7]. After carefully analyzing the spinal anatomy and clinical facts, Yu Wang et al. recently redefined the concept “lateral lumbar spinal canal” (LLSC) and creatively provided a new classification of LLSC with five different zones [8]. We found that the retrodiscal space (zone 1) and upper bony lateral recess (zone 2) were the two most common regions for occurrence of lumbar degeneration. In clinical practice, stenosis simultaneously occurring in both zones 1 and 2 were most common (43.4%) [8]. Unfortunately, endoscopic decompression for patients was difficult, even for experienced endoscopic spine surgeons, due to the complicated compressive situation.

Percutaneous endoscopic lumbar foraminoplasty was initially used to enlarge the foramen by trephine and/or high-speed drill. Thereafter, foraminoplasty was used as an efficient decompressive method for treating LSS [3, 9]. The procedure of foraminoplasty was facilitated by changing the specific location of the needle tip and trajectory of trephine to decompress different compressive pathology. Foraminoplasty was performed in order to resect the upper-ventral aspect of the superior articular process (SAP) in a classical transforaminal endoscopic spine system (TESSYS) technique [10]. However, the removed scale was not enough for stenosis patients with both zones 1 and 2 involvement; additional endoscopic dorsal decompression was required, using a high-speed drill during the operation. The disadvantages were obvious: increased surgery time, additional risk of iatrogenic nerve root/dural sac injury, and particularly postoperative low back pain (LBP) and potential spinal instability caused by excessive removal of SAP.

To realize more accurate decompression and minimize the destruction of the facet joints, we creatively applied the accurate double percutaneous lumbar foraminoplasty and PELD (DPLF–PELD) by separately removing the upper-medial-ventral portion of the facet joint and lower medial-ventral part of SAP for stenosis patients with involvement of both zones 1 and 2. In our previous retrospective study in 2016, 29 patients achieved satisfactory clinical outcomes, with an excellent rate of 93.3% by using this innovative technique [11]. The present study was prospectively designed to re-evaluate the clinical outcomes of recent similar patients by using DPLF–PELD with the help of our specially designed depth-limited instruments. Technique notes and short-term outcomes are included in this report.

## Materials and methods

### Study design

This prospective cohort study was approved by the Ethics Committee of West China Hospital, Sichuan University and was registered with the Chinese Clinical Trial Registry (ChiCTR1800019551). The study was conducted in accordance with the Declaration of Helsinki. Written informed consent was obtained from all participants prior to surgery.

Here, except for 2 patients who refused to participate in the study and 2 patients with controversial diagnosis, 69 patients with single-level LLSC stenosis, simultaneously occurring in both zones 1 and 2 from November 2018 to April 2019, were enrolled. All of the included patients underwent DPLF–PELD performed by one endoscopic spine surgeon (KQQ). Table 1 presents the characteristics of 69 patients. LBP, muscle weakness of the lower limbs, extremity radiating pain with/without gluteal pain, and neurogenic intermittent claudication were observed in 5 (7.2%), 1 (1.4%), 62 (89.8%), and 53 (76.8%) patients, respectively. No patients had a history of surgeries.

**Table 1:** Characteristics of patients undergoing accurate double percutaneous lumbar foraminoplasty (DPLF) and percutaneous endoscopic lumbar decompression (PELD)

**Patient data**	
**Age at presentation, yrs**	66.1 ± 7.5^a^(range, 42–91 years)
**Male gender**	40 (57.9%)
**Occupation**	
Sedentary	18 (26.1%)
Light work	30 (43.4%)
Heavy manual work	21 (30.5%)
Duration of symptoms, mo	20.9 ± 5.6^a^ (range, 4–90 months)
**Level of involvement**	
L3/4	0 (0%)
L4/5	57 (82.6%)
L5/S1	12 (17.4%)
**Side of LLSC stenosis**	
left	39 (56.5%)
right	30 (43.5%)
**Patients with comorbidities**	
Diabetes mellitus	27 (39.1%)
Smoking	24 (34.8%)
Alcohol consumption	12 (17.4%)
Osteoporosis	22 (31.8%)
Hypertension	27 (39.1%)
Use of antidepressants	1 (1.4%)
**Physical treatment and medications**	
Steroid intake	19 (27.5%)
Nerve blocks/epidural blocks	11 (15.9%)

^a^Data represented as mean (±standard deviation)

### Inclusion and exclusion criteria

**Fig. 1:**
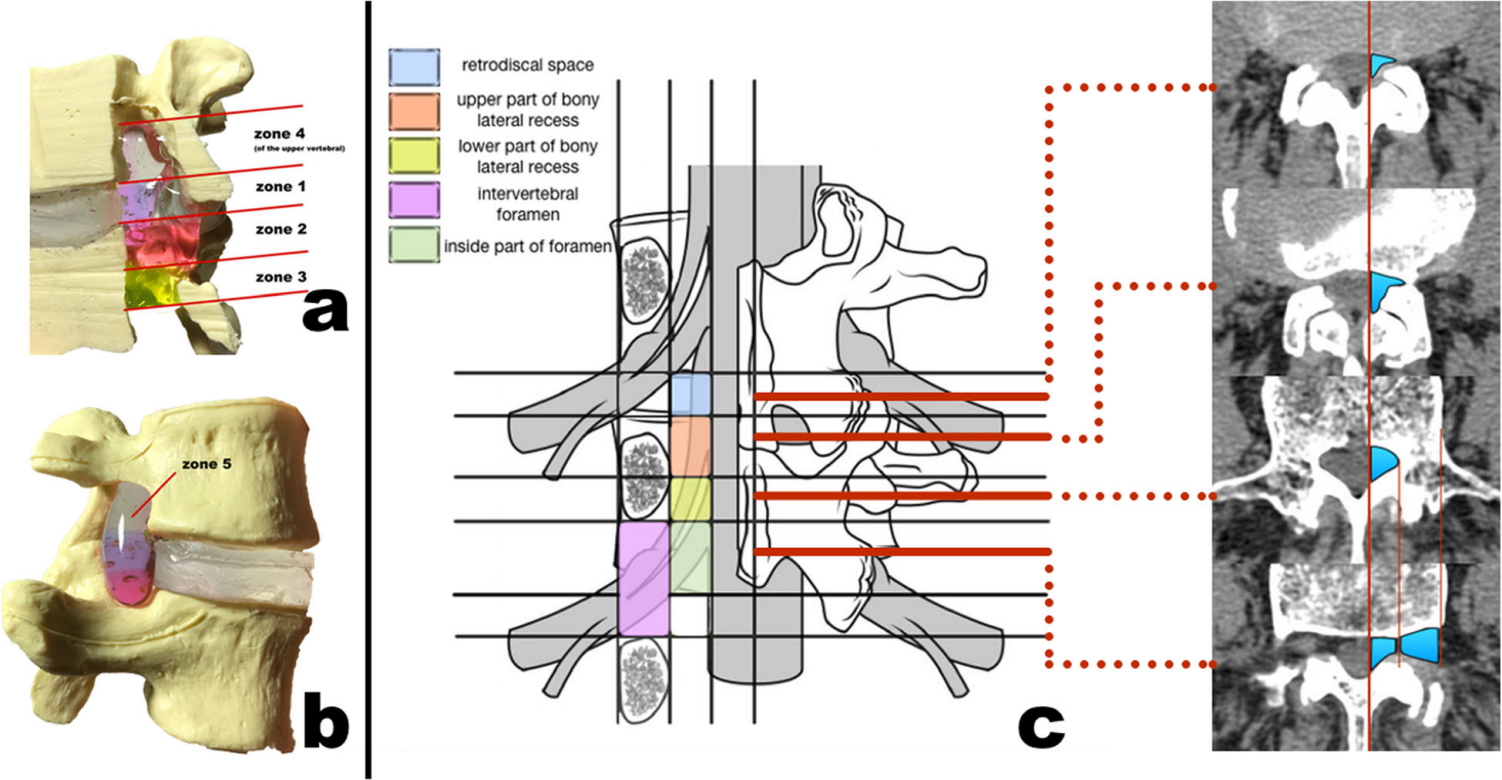
Five zones of the lateral lumbar spinal canal (LLSC) divided by accurate boundaries. **a,b** Different zones shown in an artificial model in both medial and lateral views. **c** schematic diagram of the five zones. The boundaries of each zone is described in the text. The right four axial CT scanning image shows the different views of cross-sections (the red solid lines) and the labeled regions correspond to each zone

The study included the following patients: (1) Those who manifested a single nerve root symptom, such as single-side extremity pain, numbness, or weakness with or without LBP. (2) Those who possessed full preoperative radiological information. The method of distinguishing the stenotic zone has been described in a previous study (Fig. 1 [8]. Stenosis in zone 1 was diagnosed by sagittal T2-weighted MRI scans through the paracentral region: the anteroposterior distance measured less than 1 mm. Stenosis in zone 2 was diagnosed by axial bone window CT scans, which showed that the anteroposterior distance in the lateral recess region was less than 3 mm. The radiological diagnosis should be related to clinical symptomatology. The preoperative blocking of the nerve root could be applied in some intractable cases. (3) Those who presented with obvious symptoms (preoperative leg pain visual analog score [VAS] score over 6) after over 3 months of ineffective conservative treatment. (4) Those who provided informed consent for our study and agreed to attend all required follow-up visits.

The study excluded the following patients: (1) those with lumbar segmental instability indicated by preoperative lumbar flexion-extension x-rays; (2) those who were diagnosed with lumbar central canal stenosis; (3) those who were diagnosed as having a pure lumbar disk herniation; (4) those with a high-grade lumbar spondylolisthesis with multilevel spinal stenosis or other deformity; (5) those exhibiting a high iliac crest, with the peak of the iliac crest surpassing the lower quarter of the L4 vertebral body, hindering puncture at L5/S1; and (6) those with any type of surgical contraindication.

In order to minimize the selection bias, in addition to strictly grasping the inclusion and exclusion criteria, three observers (WY, DMY and WH) simultaneously judged the stenosis area of each patient through preoperative CT and MRI imaging data. Each observer was blinded to the patient. The patient will be included in the study only when all three observers judged that the stenosis occurred in both zones 1 and 2. This method is similar to the reliability test in our previous study [8].

### Special surgical tools

**Fig. 2:**
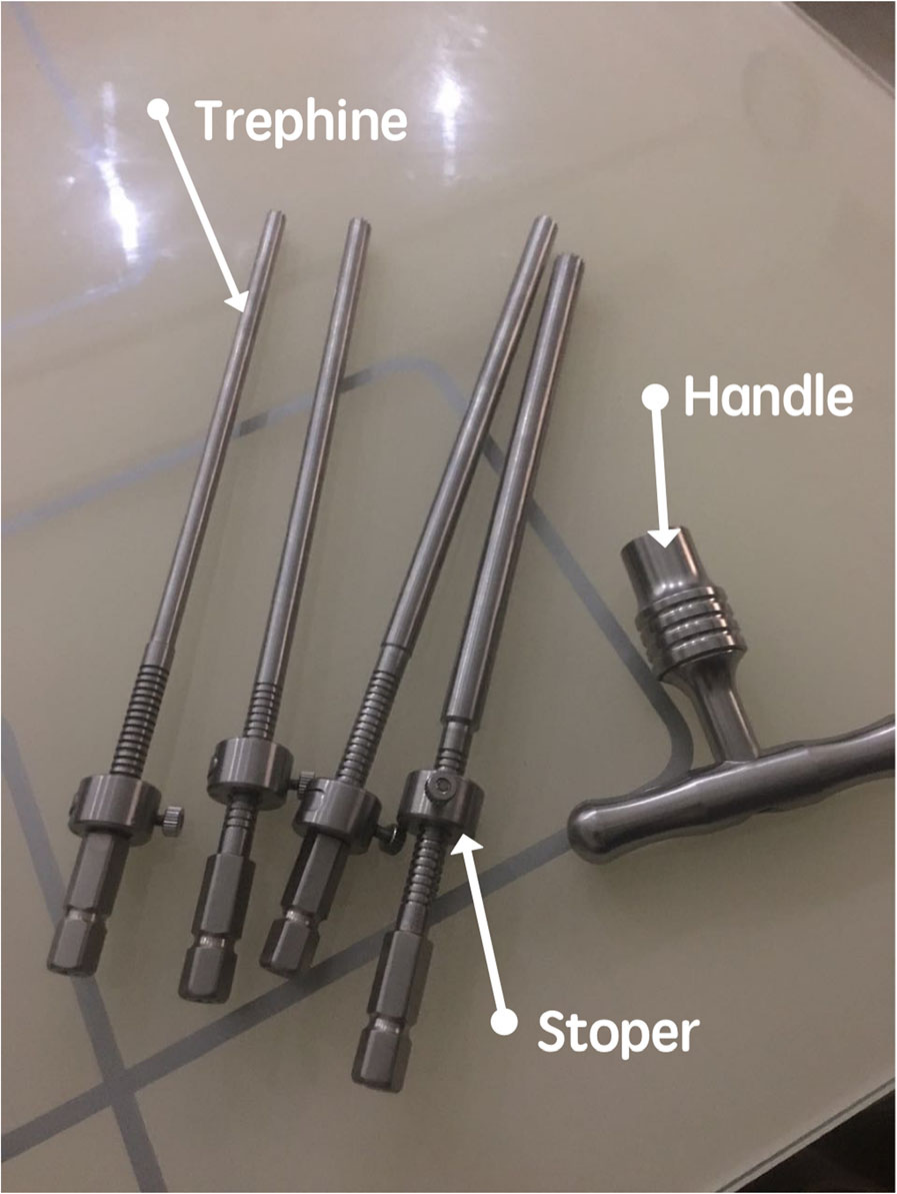
The specially designed depth-limited trephine

Specially designed depth-limited trephine for foraminoplasty (ZL 201621149959.2): consisted of a trephine, handle, and stopper (Fig. 2). The tools has been described in detail in our previous research [12].

### Surgical techniques

**Fig. 3:**
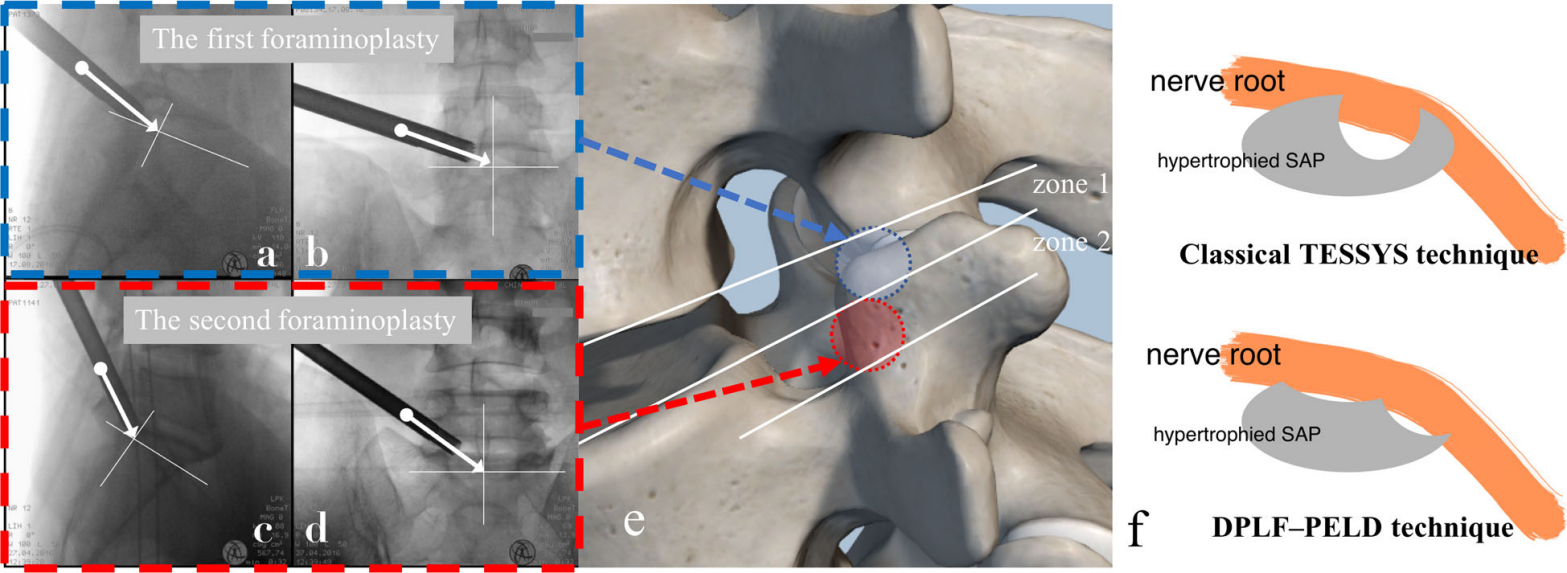
**a, b** Schematic diagram of the inclination of the trephine trajectory in lateral and AP (anterior-posterior) views in the first foraminoplasty. **c, d** Schematic diagram of the inclination of the trephine trajectory in lateral and AP views in the second foraminoplasty. The cross point of the white solid line is described in the text. **e** schematic diagram of the two-time percutaneous lumbar foraminoplasty procedure. **f** schematic diagram of the difference between classical TESSYS technique and double percutaneous lumbar foraminoplasty and PELD (DPLF–PELD) technique

All DPLF–PELD procedures employed by the author were essentially a classic THESSYS technique popularized by Hoogland [9]. The procedure of inserting needle, guide wire, obturator and trephine protection tube was performed as we previously reported [12]. A trephine protection tube (6.5-, 7.5-, or 8.5-mm-diameter) was introduced over the obturator until it was situated in the proper position. The depth-limited trephine designed by us (6.5-, 7.5-, or 8.5-mm-diameter, selected based on pathologic conditions) was used to perform two-time foraminoplasty, which was facilitated by changing the trajectory of the trephine, to aim for different compressive portions. The details of the two foraminoplasty procedures are shown in Table 2 and Fig. 3.

**Table 2:** Details of the two foraminoplasty procedure

	Target region	The inclination of the trephine		Removed section	The depth of foraminoplasty
		In lateral view	In AP view		
**The first foraminoplasty**	Retrodiscal space (Zone 1)	From the tip of superior articular process (SAP) to the posterior rim of the upper endplate of distal vertebral	From the tip of SAP to midpoint of upper endplate of distal vertebral body	Upper-medial-ventral part of facet joint which comprise tip and upper-ventral part of SAP, a part of inferior articular process (IAP) and a small ventral part of laminar.	Limited to 10–12 mm controlled by the special designed trephine
**The second foraminoplasty**	Upper bony lateral recess (zone 2)	From the tip of SAP to the cross-point of middle pedicular line and the posterior surface of vertebral body	From the tip of SAP to midpoint of middle pedicular line	Lower medial-ventral part of SAP	Limited to 12–14 mm controlled by the special designed trephine

In the first foraminoplasty, the scale of the resection could be slightly adjusted, based on different pathologic conditions. After the first foraminoplasty, a radiofrequency probe was endoscopically used (working tube with an elevator tip, ID 7.2 mm, OD 8.0 mm, and L178 mm; spinal endoscope, 30° direction of view, WC 3.75 mm, OD 6.3 mm, and WL 181 mm) to control bleeding and to adequately expose bony structures by resecting any adherent soft tissue. The margin of exposure should run from the upper-ventral surface of the SAP to the lower-ventral surface of the SAP and upper surface of the pedicle. Next, a 1.5-mm Kirschner wire was inserted into the aiming site. After removing the spinal endoscope, the second foraminoplasty was then performed, after positioning the trephine protection tube over the Kirschner wire and adjusting its tip to embrace the ventral-basal aspect of the SAP. In some severe stenosis cases, to prevent injury to the nerve roots, we only inserted the trephine into the three quarters of the SAP, thus breaking the involved SAP, instead of performing a complete resection by the trephine. For the two foraminoplasty procedures, the trephine needed to be underdraught, thus aiming to resect more of the SAP. The order of the two steps can be adjusted according to different situations.

In the following step, the trephine protection tube was replaced with the working tube with an elevator tip. High-speed drilling was then used to resect the remaining hypertrophied SAP or IAP as needed. The working tube was adjusted to completely remove decompressive factors: the hypertrophied ligamentum flavum, facet joints, and anterior herniated disk. To reduce the recurrence rate of lumbar disk herniation (LDH), we did not perform discectomy (only decompress dorsal compressive factors) for patients whose annulus was not damaged. The compressed nerve root was decompressed and explored from the distal end to near-end, especially at the attachment point of the annulus. The surgeon could see and mobilize both the traversing nerve root and the exiting nerve root under endoscopic visualization. Free movement of the dural sac and nerve root could be a sign of complete decompression. Epidural bleeding was controlled with a radiofrequency probe under saline irrigation.

For each operation duration, times of intraoperative C-arm fluoroscopy use and any complications were recorded. Every patient was asked to wear lumbar protection devices for 2-4 weeks after the operation, and to take muscle function exercise the initial 2 weeks following the surgery.

### Outcome assessment

Outcomes were evaluated via follow-up interviews (WY) who was blinded to each patients at 3 months and a final follow-up post-surgery. We used LBP, leg pain VAS, and Oswestry disability index (ODI) to evaluate the outcomes of surgery. Function outcomes were assessed using the modified Macnab criteria [13]. All patients routinely underwent 3D-reconstructive CT scans 2 days after the operation as well as MRI and CT scans after 3 months to confirm complete decompression. In the final follow-up, patients underwent CT to confirm no recurrence of LLSC stenosis, and flexion-extension x-rays to observe for lumbar stability. All patients’ postoperative radiological exams are permitted to be discharged.

### Sample size calculation

Calculation of the required sample size for this study is not constructive. This study is a case series based on the assumption that for introducing and acquiring experience in a modified operative technique. We ultimately included 69 cases in the study though a sample size of 30 patients is enough according to previous reports [14].

### Statistical analysis

Statistical analysis was performed with SPSS 23 software (SPSS Inc., Chicago, IL). Preoperative and postoperative (three-month and final follow-up) VAS and ODI scores (calculated as mean ± standard deviations) were analyzed with analysis of variance (ANOVA). Here, *p* < 0.05 was considered the threshold for significance.

## Results

### Clinical outcomes

**Fig. 4:**
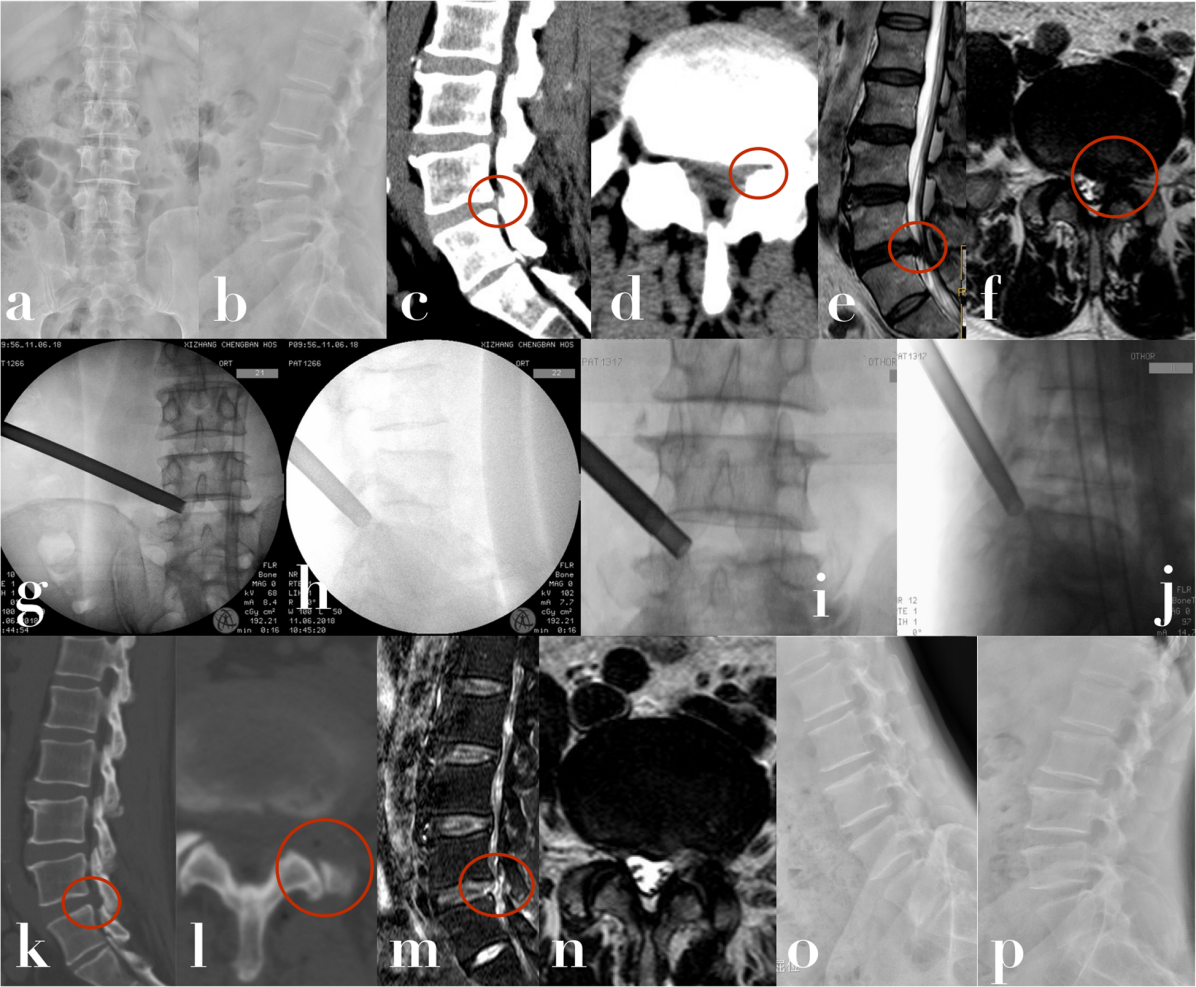
The patient complained of severe left radicular pain for 12 months. He could not walk for 3 months due to severe left buttock and leg pain. Left L4/5 LLSC stenosis in both zones 1 and 2 was confirmed. We confirmed the totally decompression by postoperative CT and MRI. The leg pain was relieved immediately after the operation. No lumbar instability was indicated in the final follow-up. **a, b** Preoperative X-ray in AP and lateral view. **c** Preoperative sagittal CT scans indicated stenosis of the retrodiscal space (zone 1) on the left at L4/5 (red circle). **d** Preoperative axial CT scans indicated upper bony lateral recess (zone 2) stenosis on the left at L4/5 (red circle). **e, f** Preoperative sagittal and axial T2-weighted MRI scans showing the L4/5 left zone 1 stenosis caused by a lumbar disk bulge anteriorly, and hypertrophied, curled ligamentum flavum posteriorly (red circle). **g, h** Fluoroscopy during surgery demonstrates the trajectory of the trephine in the first foraminoplasty in the AP and lateral views. **i, j** Fluoroscopy during the operation shows the trajectory of the trephine in the second foraminoplasty in the AP and lateral views. **k, l** Three months postoperative sagittal and axial CT bony-window scans clearly demonstrate complete decompression of zones 1 and 2. **m, n** Three months postoperative sagittal and axial T2-weighted MRI scans indicate that the nerve root was decompressed without recurrence. **o, p** Postoperative flexion-extension x-rays at the final follow-up confirm that no lumbar instability occurred

All patients successfully underwent DPLF–PELD without hematoma formation, a change to open surgery, or any nerve root injuries. Leg pain was immediately eased after the operation. The mean follow-up period was 13 months (range, 8–17 months). In the final follow-up, leg pain VAS was 0.75 ± 0.63 which was significantly decreased compared to 7.05 ± 1.04 preoperatively. The preoperative LBP VAS score was 1.34 ± 0.48, which also significantly decreased to 0.93 ± 0.31 in final follow-up (*p* < 0.05). in addition, the excellent rates among involved patients was 94.2% in the final follow-up results. All clinical outcome results are shown in Table 3. Two patients had dural tear complications. They were cured after a conservative treatments. There was one patient whose preoperative symptoms were not relieved by the surgery. The postoperative CT scan illustrated a small separated bony segment that had moved into the spinal canal. After 2 months of conservative treatment, the symptoms became aggravated, and we performed open surgery. The symptoms completely disappeared immediately. Two patients complained of moderate postoperative LBP without lumbar muscle weakness, which disappeared after conservative treatment. All three-month postoperative CT and MRI exams confirmed that the compressive factors were completely removed, by showing complete removal of the dorsal hypertrophied SAP on CT, and CSF filling around the compressed nerve root on MRI. The flexion-extension x-rays and CT at final follow-up indicated no recurrence or lumbar segmental instability. A case presentation is shown in Fig. 4.

**Table 3:** Clinical outcomes of patients with precise safety decompression via accurate double percutaneous lumbar foraminoplasty (DPLF) and percutaneous endoscopic lumbar decompression (PELD)

**The mean operative duration time, min**	63.2 (range, 30–110 min)	
**The mean length of hospital stay, day**	4.52 (range, 3–9 days)	
**the mean times of intraoperative C-arm fluoroscopy use**	13.8 (range, 5–41)	
**VAS (leg pain/LBP)**	**Mean (SD)**	**Significance level**
Pre op	7.05 ± 1.04/1.34 ± 0.48	
Post-op 3 mo	1.03 ± 0.79/1.02 ± 0.28	*P* < 0.05*/ *P* > 0.05
Final follow-up	0.75 ± 0.63/0.93 ± 0.31	
**ODI**	**Mean (SD)**	**Significance level**
Pre op	69.8 ± 9.05	
Post-op 3 mo	20.3 ± 5.52	*P* < 0.05*
Final follow-up	19.6 ± 5.21	
**Subjective outcomes**^a^		
Excellent	49	
Good	16	
Fair	3	
Poor	1	
**Satisfactory (excellent or good) results**	65/69 (94.2%)	

^a^Macnab criteria

*Paired Student test

## Discussion

The term “lateral lumbar spinal canal” was first introduced by Lee et al. in 1988, and was divided into entrance, middle, and exit zones [5]. However, problems were noted with the label, such as ambiguous borders of each zone and improper names [15]. Prior to our systemically defining LLSC, and providing a classification, there was no universally accepted definition of LLSC, including the lateral recess region [8]. We found that the retrodiscal space (zone 1) and upper bony lateral recess (zone 2) are the two most common regions in which lumbar degenerative changes occur. As we analyzed [8], zone 1 is surrounded by soft tissue whose dorsal compressive element was the ligamentum flavum and joint capsules; zone 2 was formed by tricortical bony structures whose compressive element was hypertrophied SAP. Consequently, regardless of LSS occurring in zone 1 and/or 2, to ensure the effectiveness of surgery, accurate and complete surgical decompression is important. However, for LLSC stenosis patients with the involvement of both zones 1 and 2, complete surgical decompression necessitates higher requirements. Conventional open surgery can adequately treat LLSC stenosis patients via a resecting laminectomy and medial arthrotomy, however, the drawback is obvious: a longer operation time, lengthier recovery period, and more complications [16].

In recent years, PELD has greatly developed, making revolutionary progress [17–19]. The application of foraminoplasty greatly expanded the indications for PELD in treating LSS [3, 20, 21]. Various foraminoplasty methods are available by adjusting the location of needle tip and the trajectory of trephines, achieving the purpose of decompression on specific targets. For example, the classical TESSYS technique popularized by Hooglan et al. first introduced the foraminoplasty procedure by resecting the upper-ventral aspect of the SAP [22]. Afterwards, variations of the TESSYS technique were created, aiming to remove the lower-ventral portion of an hypertrophied SAP in lateral recess stenosis patients [9, 23]; recently, our team created trans-articular and trans-isthmus approaches as foraminoplasty methods to treat central/paracentral and high upper-migrated LDH patients, respectively [12, 24].

However, endoscopic decompression toward zones 1 and 2 required different foraminoplasty targets (Fig. 3). It was very difficult to realize full-course decompression via single foraminoplasty with a classical TESSYS technique, without the use of an endoscopic high-speed drill. The frequent usage of a high-speed drill is bound to cause additional length of surgery, a higher risk of iatrogenic nerve root/dural sac injury and excessive resection of the SAP, which can cause potential postoperative LBP and lumbar segmental instability [3]. Therefore, after carefully analyzing the anatomical, pathological, and biomechanical features of LLSC mentioned in the published study [8], and combined with extensive endoscopic surgical practice, we creatively designed precise safety decompression via DPLF–PELD, which performed programed and accurate foraminoplasty toward zones 1 and 2 separately. The advantages were obvious. On the one hand, the programed operation greatly improved decompression efficiency and accuracy. This guaranteed the full-course and complete decompression of the two regions with a shorter timeframe. On the other hand, more of the normal SAP can be retained because most principle compression in zones 1 and 2 would be accurately resected in the two-time programed foraminoplasty procedures. This can largely prevent the occurrence of the postoperative LBP and potential lumbar segmental instability, as showed in Fig. 3f.

Of the 69 patients in our study, the preoperative leg pain VAS score was 7.05 ± 1.04, which decreased to 1.03 ± 0.79 postoperatively (*p* < 0.05). Additionally, we did not find the increasing postoperative LBP VAS score in our group (*p* > 0.05). This indicates that our modified technique did not necessarily increase iatrogenic postoperative LBP, which was an additional benefit as compared to conventional open surgery. We owing this to the advantages of minimally facet joint damage. The final follow-up results revealed an excellent rates (94.2%), which was similar to conventional microsurgical techniques [25],and was higher than other endoscopic techniques: 82% in Kambin [26], 85% in Lewandrowski [23] and 89.2% in Yeung [27]. No incomplete decompression, nerve root injury, or other complication appeared. No recurrence and segmental instability was observed in any patient during the follow-up period. We propose that the good clinical outcome was a result of using the classification, confirming compressive factor preoperatively, using TPLF–PELD realizing full-course complete decompression, and avoiding unnecessary resection of SAP intraoperatively. Furthermore, our specially designed the depth-limited trephine effectively guaranteed the safety of the procedures. For those severe stenosis patients, the nerve root was tightly compressed by hypertrophied facet joint. The nerve root may be easily injured by excessive advance of the trephine without depth limitation.

Among all 69 patients, only one suffered a severe postoperative complication, consisting of small separated bony material remaining in the spinal canal, which was subsequently removed by open surgery 2 months later. This was caused by insufficient experience in the early period. Additionally, there were two cases of intraoperative dural tear, cured by conservative treatment. We attributed these occurrences to the severe adhesion between the dural sac and surrounding bony structures caused by long-term stenotic changes. The complication rate was 4.3%, which was apparently lower than in other studies ranging from 5.5–13.2% [9, 26, 27]. The above-mentioned results proved the effectiveness and rationality of the DPLF–PELD in treating LLSC stenosis in both zones 1 and 2.

We were also concerned about the effect on postoperative lumbar segmental stability, following removal of a portion of the facet joint. Although it has been demonstrated that facetectomy decreases the stiffness and increases the mobility of the spinal motion segment in all modes of loading [28, 29], there is still no evidence that injured or damaged facet joints consequently induce the mechanical instability of the spine [30]. Furthermore, our technique only resects a small segment of the facet joint, comprising less than 10–20% of the entire facet joint. Osman studied the pathoanatomic and flexibility changes after posterior and transforaminal decompression in a cadaver biomechanical study [31], which even much more destruction than ours. No flexibility change or instability was noted, identical results as obtained in our previous reports [8, 11, 12, 24]. In our study, we designed lumbar dynamic position x-rays at each patient’s final follow-up. No postoperative iatrogenic segmental instability was observed.

The limitations of this study should be noted. First of all, the study was designed as a prospective self-control cohort study. We did not compare the modified DPLF–PELD to other technique. The lack of a control group was the primary limitation of our study. Observations of the advantages of DPLF-PELD compared with other method was needed in further study. Besides, the small sample size and a short follow-up period. In addition, TPLF-PELD has a steep learning curve and relative narrow indication: the surgery is only suited to simple single-level LLSC stenosis patients who have both zones 1 and 2 involvement. The number of patients who qualified for the study was therefore quite limited, and further studies are needed.

## Conclusion

DPLF–PELD is a minimally-invasive, effective, and safe surgical method that can well treat LLSC stenosis patients whose stenotic region has occurred in both zones 1 and 2, with the advantages including less damage to lumbar spine anatomy, a lower complication rate, and good short-term clinical outcomes.


**Additional file 7.**


## Supplementary Information

**Additional file 1.**

**Additional file 2.**

**Additional file 3.**

**Additional file 4.**

**Additional file 5.**

**Additional file 6.**

## Data Availability

The datasets used and/or analysed during the current study are available from the corresponding author on reasonable request. Any researcher interested in finding this dataset can submit an application letter to the corresponding author. Please contact the Email (kqqspine@126.com) for further assistance.

## References

[1] Lurie J, Tomkins-Lane C (2016). Management of lumbar spinal stenosis. BMJ.

[2] Inoue G, Miyagi M, Takaso M (2016). Surgical and nonsurgical treatments for lumbar spinal stenosis. Eur J Orthop Surg Traumatol.

[3] Ahn Y (2014). Percutaneous endoscopic decompression for lumbar spinal stenosis. Expert Rev Med Devices.

[4] Issack PS, Cunningham ME, Pumberger M, Hughes AP, Cammisa FP (2012). Degenerative lumbar spinal stenosis: evaluation and management. J Am Acad Orthop Surg.

[5] Lee CK, Rauschning W, Glenn W (1988). Lateral lumbar spinal canal stenosis: classification, pathologic anatomy and surgical decompression. Spine (Phila Pa 1976).

[6] Crock HV (1981). Normal and pathological anatomy of the lumbar spinal nerve root canals. J Bone Joint Surg Br.

[7] Lassale B, Morvan G, Gottin M (1984). Anatomy and radiological anatomy of the lumbar radicular canals. Anat Clin.

[8] Wang Y, Dou Q, Yang J, Zhang L, Yan Y, Peng Z, Guo C, Kong Q (2018). Percutaneous endoscopic lumbar decompression for lumbar lateral Spinal Canal stenosis: classification of lateral region of lumbar Spinal Canal and surgical approaches. World Neurosurg.

[9] Li ZZ, Hou SX, Shang WL, Cao Z, Zhao HL (2016). Percutaneous lumbar foraminoplasty and percutaneous endoscopic lumbar decompression for lateral recess stenosis through transforaminal approach: technique notes and 2 years follow-up. Clin Neurol Neurosurg.

[10] Ahn Y (2014). Percutaneous endoscopic decompression for lumbar spinal stenosis. Expert Rev Med Dev.

[11] Wang Y, Kong Q, Song Y (2017). Short-term effectiveness of accurate decompression via foraminoplasty in treatment of lumbar lateral recess stenosis. Zhongguo Xiu Fu Chong Jian Wai Ke Za Zhi.

[12] Wang Y, Yan Y, Yang J, Zhang L, Guo C, Peng Z, Wu H, Zhang D, Kong Q. Outcomes of percutaneous endoscopic trans-articular discectomy for huge central or paracentral lumbar disc herniation. Int Orthop. 2018. 10.1007/s00264-018-4210-6. 10.1007/s00264-018-4210-6 30374637

[13] Macnab I (1971). Negative disc exploration. An analysis of the causes of nerve-root involvement in sixty-eight patients. J Bone Joint Surg Am.

[14] Zeiders GJ, Patel MK (2008). Management of unstable elbows following complex fracture-dislocations--the "terrible triad" injury. J Bone Joint Surg Am.

[15] Wang Y, Kong Q (2017). To the editor. Spine (Phila Pa 1976).

[16] Ang CL, Phak-Boon Tow B, Fook S, Guo CM, Chen JL, Yue WM, Tan SB (2015). Minimally invasive compared with open lumbar laminotomy: no functional benefits at 6 or 24 months after surgery. Spine J.

[17] Kambin P (1992). Arthroscopic microdiscectomy. Arthroscopy.

[18] Zheng C, Wu F, Cai L (2016). Transforaminal percutaneous endoscopic discectomy in the treatment of far-lateral lumbar disc herniations in children. Int Orthop.

[19] Wang K, Hong X, Zhou BY, Bao JP, Xie XH, Wang F, Wu XT (2015). Evaluation of transforaminal endoscopic lumbar discectomy in the treatment of lumbar disc herniation. Int Orthop.

[20] Yeung AT (2000). The evolution of percutaneous spinal endoscopy and discectomy: state of the art. Mt Sinai J Med.

[21] Yeung AT (2007). The evolution and advancement of endoscopic Foraminal surgery: one Surgeon's experience incorporating adjunctive Techologies. SAS J.

[22] Hoogland T, Schubert M, Miklitz B, Ramirez A (2006). Transforaminal posterolateral endoscopic discectomy with or without the combination of a low-dose chymopapain: a prospective randomized study in 280 consecutive cases. Spine.

[23] Lewandrowski K-U (2014). &apos;&apos;outside-in&apos;&apos; technique, clinical results, and indications with Transforaminal lumbar endoscopic surgery: a retrospective study on 220 patients on applied radiographic classification of Foraminal spinal stenosis. Int J Spine Surg.

[24] Yan Y, Wang Y, Yang J, Wu H, Zhang L, Peng Z, Guo C, Kong Q (2018). Percutaneous endoscopic lumbar discectomy for highly Upmigrated disc herniation through the Transforaminal isthmus Plasty approach. World Neurosurg.

[25] Ruetten S, Komp M, Merk H, Godolias G (2008). Full-endoscopic interlaminar and transforaminal lumbar discectomy versus conventional microsurgical technique: a prospective, randomized, controlled study. Spine (Phila Pa 1976).

[26] Kambin P, Casey K, O'Brien E, Zhou L (1996). Transforaminal arthroscopic decompression of lateral recess stenosis. J Neurosurg.

[27] Yeung AT, Tsou PM (2002). Posterolateral endoscopic excision for lumbar disc herniation: surgical technique, outcome, and complications in 307 consecutive cases. Spine (Phila Pa 1976).

[28] Tender GC, Kutz S, Baratta R, Voorhies RM (2005). Unilateral progressive alterations in the lumbar spine: a biomechanical study. J Neurosurg Spine.

[29] Abumi K, Panjabi MM, Kramer KM, Duranceau J, Oxland T, Crisco JJ (1990). Biomechanical evaluation of lumbar spinal stability after graded facetectomies. Spine (Phila Pa 1976).

[30] Jaumard NV, Welch WC, Winkelstein BA (2011). Spinal facet joint biomechanics and mechanotransduction in normal, injury and degenerative conditions. J Biomech Eng.

[31] Osman SG, Nibu K, Panjabi MM, Marsolais EB, Chaudhary R (1997). Transforaminal and posterior decompressions of the lumbar spine. A comparative study of stability and intervertebral foramen area. Spine (Phila Pa 1976).

